# Supervised Clustering of Adipokines and Hormonal Receptors Predict Prognosis in a Population of Obese Women with Type 1 Endometrial Cancer

**DOI:** 10.3390/ijms18051055

**Published:** 2017-05-13

**Authors:** Jennifer Uzan, Enora Laas, Issam Abd Alsamad, Dounia Skalli, Dhouha Mansouri, Bassam Haddad, Cyril Touboul

**Affiliations:** 1 Department of Obstetrics and Gynecology, Centre Hospitalier Intercommunal de Créteil, 40 Avenue de Verdun, 94000 Créteil, France; jen.uzan@gmail.com (J.U.); dounia.skalli@chicreteil.fr (D.S.); bassam.haddad@chicreteil.fr (B.H.); 2 Department of Pathology, Centre Hospitalier Intercommunal de Créteil, 40 Avenue de Verdun, 94000 Créteil, France; issam.abdalsamad@chicreteil.fr (I.A.A.); dhouha.mansouri@chicreteil.fr (D.M.); 3 Department of Surgery, Institut Curie, 26 rue d’Ulm, 75005 Paris, France; enolaas@gmail.com; 4 INSERM/Paris 7 U965 “Carcinose, Angiogénèse-Recherche Translationnelle”, Centre Hospitalier Universitaire Lariboisière, Assistance Publique des Hôpitaux de Paris (AP-HP), 2 rue Ambroise Paré, 75010 Paris, France

**Keywords:** obesity, endometrial cancer, adipokine, prognosis

## Abstract

Obesity is a major risk factor for endometrial cancer (EC). Yet, its impact on prognosis is controversial. Obesity is associated with metabolic and hormonal dysregulation as well as adipokines increase. The aim of this study was to characterize the expression of biological factors related to obesity within the tumor and evaluate their impact on prognosis. One hundred and thirty-six patients, including 55 obese patients, with endometrioid type I EC operated by total hysterectomy were included in this retrospective study conducted in a Tertiary teaching hospital between 2000 and 2013. Immunohistochemistry (IHC) study was performed on type I EC tumor samples using five adipokines (SPARC, RBP4 (Retinol Binding Protein 4), adiponectin, TNF α, IL-6) and hormonal receptors (estrogen receptor and progesterone receptor). Supervised clustering of immunohistochemical markers was performed to identify clusters that could be associated with prognostic groups. The prognosis of the obese population was not different from the prognosis of the general population. Adipokine expression within tumors was not different in these two populations. In obese population, we found three clusters where co-expression was associated with a recurrence group in comparison with a non-recurrence group and four clusters where co-expression was associated with the high risk FIGO (International Federation of Gynecology and Obstetrics) stage I group in comparison of low risk FIGO stage I group. While obesity does not appear as a prognostic factor in endometrioid type I EC, the co-expression of biological factors in IHC on hysterectomy specimens allowed to distinguish two prognostic groups in obese population.

## 1. Introduction

Obesity, defined as a body mass index (BMI) higher than 30 kg/m^2^, is a major risk factor for endometrial cancer (EC) [1,2] especially for type 1 or endometrioid type [3]. In the United States, the risk of death with EC is multiplied by 2.53 for a BMI between 30 and 34 kg/m^2^, and multiplied by 6.25 for a BMI higher than 40 kg/m^2^ [2,4]. However, no impact of obesity has been found on progression-free survival (PFS) or overall survival (OS) in EC [2,5,6,7]. Moreover, studies focusing on the impact of obesity on prognostic factors such as histologic grade, FIGO stage, or lymphovascular space involvement (LVSI) have reported controversial results [5,6]. The impact of obesity on prognosis would be clinically relevant by the consecutive increased aggressiveness of the treatments, whereas these obese patients are more likely to have higher surgical and adjuvant treatment morbidities [8,9].

Obesity is biologically associated with metabolic and hormonal dysregulation that could be implied in endometrial carcinogenesis pathways. Excess free fatty acids from excess adipose tissue contribute to hyperinsulinemia, which directly stimulates the proliferation of endometrial cells and inhibition of apoptosis in vitro [10] or indirectly stimulates hyperestrogenia [11]. Indeed, excess adipose tissue promotes androgen secretion and aromatization in estrogens and causes hyperestrogenia that promotes endometrial mitotic rate [12] and atypical hyperplasia (AH), a well known precursor of type 1 EC [13]. However, the prognostic relevance of Progesterone Receptors (PR) and Estrogen Receptors (ER) expression in endometrioid carcinoma is controversially discussed [14,15,16,17].

Moreover, excess adipose tissue secretes cytokines named adipokines, which have modified serum levels in obese patients. Tumor necrosis factor α (TNF α) and Interleukin-6 (IL-6) are produced by macrophages, endothelial cells, and pre-adipocytes in excess adipose tissue [18,19], Secreted Protein Acidic and Rich in Cysteine (SPARC) is an extracellular matrix protein secreted in adipose tissue [20], and Retinol-Binding Protein 4 (RBP4) is a circulating adipokine secreted in adipose tissue [21]. These four adipokines have elevated serum levels in obese patients. On the contrary, adiponectin, produced by adipocytes, has low serum levels in obese patient [22]. Modified serum levels of these adipokines in obese patients could reflect their endocrine or paracrine action and thereby could also contribute to endometrial carcinogenesis. Several studies have reported possible associations between excess adipokines and EC. Aromatase expression in intratumoral stroma correlated positively with IL-6 expression in EC epithelial cells in immunohistochemistry (IHC) suggesting an autocrine expression of IL-6 in EC cells [23]. Also, TNF α expression in EC cells have been reported to induce invasion characteristics [24]. In vitro, adiponectin inhibits cell growth and induces apoptosis in EC cells [25] through adiponectin receptors (AdipoRs) [26] which decreased expression in EC tissue samples was associated with worse prognosis factors (higher grade, myometrial depth invasion, and lymph node metastasis) [27]. Also, a decreased expression of SPARC cDNA was found in EC tissue samples in comparison with normal endometrium tissue samples [28].

Thereby, we hypothesized that expression of these adipokines and hormonal receptors (ER and PR) were modified in EC of obese patients. Then, the first objective was to describe the IHC expression of these four adipokines and hormonal receptors in type 1 EC tissue samples of obese and non-obese patients. Also, we hypothesized that these adipokines and hormonal receptors could have a prognosis issue in obese patients with type 1 EC. The second objective of this study was to search for a prognostic immunohistochemical signature in obese patients.

## 2. Results

### 2.1. Characteristics of General and Obese Populations

The characteristics of general population (136 patients) are shown in Table 1. We considered a *p*-value <0.05 as significant. All non-significant *p*-values were >0.05. Mean BMI (SD) was 28.9 (14.8). Pelvic lymphadenectomy was performed in 110 cases (80.8%) and was associated with para aortic lymphadenectomy in 8 cases (5.8%). We distinguished one group of patients who had pelvic lymphadenectomy only to the group of patients who had pelvic and para-aortic lymphadenectomy. Patients with histological grade 1, 2 and 3 were respectively 76 (55.8%), 38 (25.7%) and 22 (18.5%). Thirty three patients (24.2%) had LVSI. One hundred and seven patients (78.7%) had FIGO stage I, 8 patients (5.8%) had FIGO stage II, 17 patients (12.7%) had FIGO stage III and 4 patients (2.8%) had FIGO stage IV. Eleven patients (8%) had positive LNs encompassing positive pelvic or para aortic LNs. Twenty-five patients (18.3%) had recurrence.

The characteristics of obese population (55 patients) are shown in Table 1. Pelvic lymphadenectomy was performed in 41 cases (74.5%) and was associated with para aortic lymphadenectomy in three cases (5.4%). Patients with histological grade 1, 2, and 3 were respectively 29 (52.7%), 17 (30.9%) and nine (16.4%). Seventeen patients (30.9%) had LVSI. Forty-three patients (78.1%) had FIGO stage I, three patients (5.4%) had FIGO stage II, eight patients (14.5%) had FIGO stage III, and one patient (2.0%) had FIGO stage IV. Five patients (9.1%) had positive LNs encompassing positive pelvic or para aortic LNs. Thirteen patients (23.6%) had recurrence. Seven patients (12.7%) had high risk FIGO stage I.

### 2.2. IHC Expression of Adipokines and Hormonal Receptors

**Figure 1:**
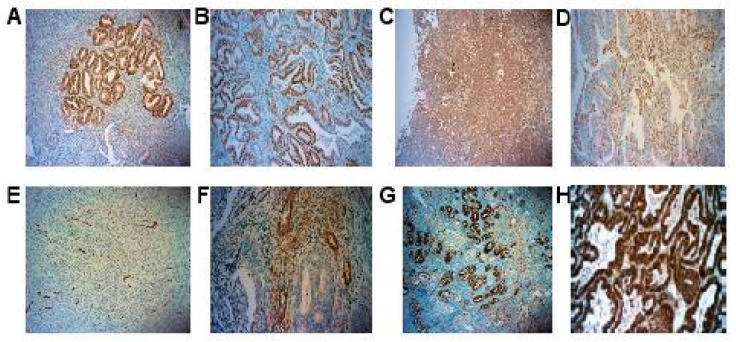
Examples of immunostaining: the positive staining was brown for each antibody; (**A**) PR immunostaining (×10); (**B**) ER immunostaining (10×); (**C**) SPARC immunostaining (10×); (**D**) stromal SPARC immunostaining (×10); (**E**) stromal adiponectin immunostaining (10×); (**F**) stromal IL-6 immunostaining (×20); (**G**) RBP4 immunostaining (10×); (**H**) TNF α immunostaining (10×).

Representative pictures of IHC are shown in Figure 1. ER and PR had nuclear immunostaining in EC cells whereas TNF α and RBP4 had cytoplasmic immunostaining in EC cells. Adiponectin had immunostaining in stromal cells and myometrial immunostaining within the invasion front of the tumor. IL-6 and SPARC had immunostaining in EC cells and stromal cells.

### 2.3. Adipokines and Hormonal Receptors Expression Were Similar in Obese and Non-Obese Population

The comparison of protein immunostaining level between obese and non-obese population using univariate analysis shown that only PR expression was significantly increased in obese population (51.64 vs. 36.67, *p* = 0.043) while all other markers were not significantly different with non-obese population.

The supervised clustering algorithm did not found any clusters of IHC markers whose co-expression could differentiate obese from non-obese populations (Figure S1).

### 2.4. Prediction of Recurrence in Obese Population

The supervised clustering algorithm found three clusters whose co-expression could differentiate a recurrence group from a non recurrence group (Table 2).

**Figure 2:**
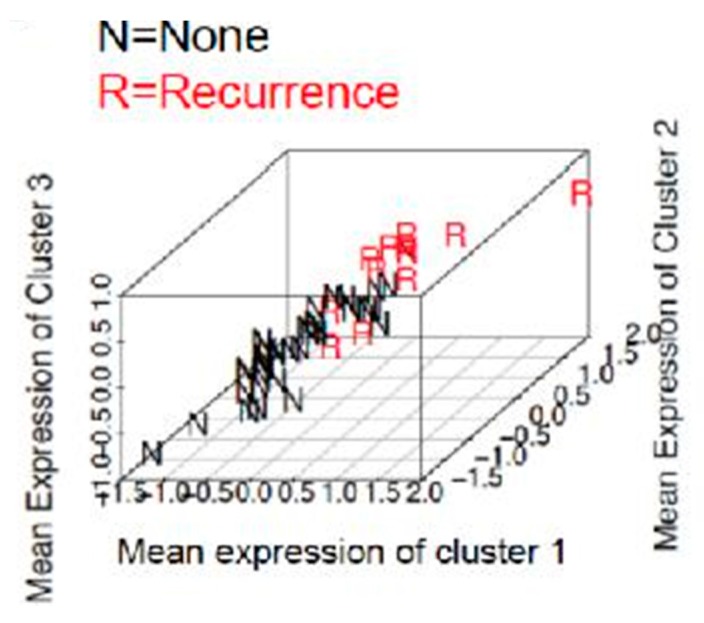
Three-dimensional representation of differentiation of recurrence and non-recurrence group according to the clusters.

In cluster 1, we found increased immunostaining of stromal IL-6 with decreased immunostaining of PR, ER and RBP4. In cluster 2, we found increased immunostaining of stromal Il-6 with decreased immunostaining of PR and RBP4. In cluster 3, we found increased immunostaining of stromal IL-6 and TNF α with decreased immunostaining of PR, ER, RBP4 and stromal adiponectin. Using these three clusters together to predict recurrence in obese population resulted in discriminating recurrence from non-recurrence with a 9% misclassification rate. Each group centroid distinguished the two groups with a high accuracy and the three-dimensional projection (Figure 2) suggests that our group centroids are suitable for predicting these two groups.

### 2.5. Prediction of A High Risk Stage I Group in Obese Population

The supervised clustering algorithm found four clusters whose co expression could differentiate high risk stage I group from low risk stage I group (Table 3).

**Figure 3:**
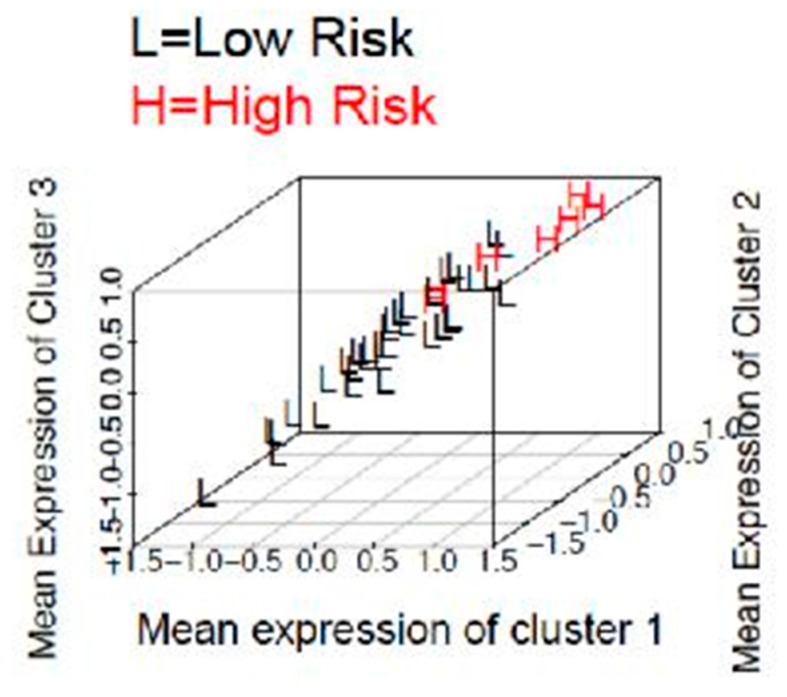
Three-dimensional representation of differentiation of high risk stage I and low risk stage I group according to the clusters.

In cluster 1, we found increased immunostaining of stromal adiponectin with decreased immunostaining of PR, ER, stromal SPARC, and RBP4. In cluster 2, we found increased immunostaining of stromal adiponectin with decreased immunostaining of stromal SPARC, PR and RBP4. In cluster 3, we found increased immunostaining of stromal adiponectin with decreased immunostaining of stromal SPARC, RBP4 and PR. In cluster 4, we found increased immunostaining of stromal adiponectin and stromal IL-6 with decreased immunostaining of stromal SPARC, RBP4, PR, ER, and SPARC. Using these four clusters together to predict risk in stage I in obese population resulted in discriminating high risk stage I from low risk stage I with a 7% misclassification rate. Each group centroid distinguished the two groups with a high accuracy and the three-dimensional projection (Figure 3) suggests that our group centroids are suitable for predicting these two groups.

### 2.6. Adipokines and Hormonal Receptors Expression Could Not Predict Prognostic Groups in Non Obese Population

The supervised clustering algorithm did not found any clusters of IHC markers whose co expression could predict LN metastasis (Figure S2), recurrence (Figure S3), or high risk stage I group (Figure S4).

## 3. Discussion

IHC analysis on type 1 EC showed that the selected adipokines are differently expressed within the tumor; staining could be located in the cytoplasm of EC cells (TNF α and RBP4), in the cytoplasm of stromal cells (adiponectin) or both in EC and stromal cells (IL-6 and SPARC). Moreover, in obese population, we found that recurrence and high risk stage I can be predicted using a combination of adipokines and hormonal receptors expressed within the tumor.

We hypothesized that adipokines and hormonal receptors expression within the tumor could be modified in obese population in comparison with non-obese population. The supervised clustering algorithm did not found clusters of IHC markers whose co-expression could differentiate obese and non-obese groups. These results could be due to the statistical tool that determines clusters of protein immunostaining that did not take into account the biological significance of each marker separately. Thereby, these results cannot exclude a difference of expression within the tumor of adipokines and steroid hormones, representative of autocrine action, as previously reported [23,24,27,28]. Moreover, these results cannot exclude a difference in systemic expression of the markers, representative of endocrine or paracrine action, in obese population in comparison with non-obese population.

Nonetheless, when we focused on obese population, we found three clusters of IHC markers whose co expression could accurately predict a recurrence group from a non-recurrence group at final histology and four clusters of IHC markers whose co-expression could accurately predict high risk stage I group from a low risk stage I group at final histology. We found that the correlation between protein immunostaining and definitive prognostic group varied when co-expression was considered. This analysis is a global prediction of recurrences or high risk patients based on a group of markers, i.e., the cluster. Thus, constitutive markers of the cluster could be implicated in the physiopathology. The supervised clustering in general population was out of the scope of this study which was focused on the relationship between obesity and adipokines. This analysis, and the analysis of the non-obese population could be the aim of another study. Moreover, as clusters enable global prediction, we hypothesized that carcinogenesis pathways were specific to the obese population. However, low number of patients in recurrence group (*n* = 13) and in high risk stage I group (*n* = 7) should lead to a careful interpretation of these results. In order to consider a large number of markers at the same time and to define groups with associated under or overexpression, new statistical methods are required. This is due to the complexity generated by a large predictor dimension (protein level) and relatively small sample size (groups), a well-known problem in genomic research where large gene expression datasets have to be analyzed. In this study, an algorithm of supervised classification was used to reveal prognostic groups of protein immunostaining. In clinical practice, IHC expression interpretation with this mathematical tool could not enable to determine a threshold of under or overexpression. These results of under and overexpression could not be used by pathologists because the variations of the level expression are standardized and must be interpreted from each other. Also, scales developed to quantify the variability of immunostaining (stromal HSCORE) were based on the H-score [29] but have not been validated and could be a limit in this study. However, the protein signature of the clusters was expressed by group centroids in a way to avoid issues linked with heterogeneity of scales.

Nevertheless, co-expression of proteins within the clusters could lead to hypothesize physiopathological features. Clusters revealed that, among the various IHC markers, the co-overexpression of TNF α and IL-6 in the stroma was associated with recurrence and high risk stage I, respectively. These results are in agreement with a study reporting that IL-6 was overexpressed in the stroma of EC and that overexpression of TNF α in EC cells was associated with poor overall survival [30].

In the present study, decreased immunostaining of ER and PR was associated with recurrence and high risk stage I as reported in previous studies focusing on steroid receptors in type 1 EC. Underexpression of ER was associated with poor overall survival [17] and underexpression of PR was associated with independent risk of recurrence [15,17]. Also, a recent study found that underexpression of ER (inferior to 30%) and underexpression of PR (inferior to 15%) was associated with positive LNs in low risk stage I EC [31].

We also found that co-underexpression of extracellular matrix protein of the tumoral microenvironnement (SPARC) was associated with high risk stage I EC. The role of SPARC has been studied in different types of cancer. In vitro, SPARC increases breast and prostate cancer cells migration and invasion [32]. In colorectal cancer, where obesity is also a well-known risk factor, stromal SPARC underexpression in IHC is associated with poor overall survival and poor progression free survival [33]. Moreover, SPARC was preferentially expressed in the stroma of EC [34]. 

Expression of adiponectin was contradictory; stromal adiponectin was underexpressed in the clusters that discriminates recurrence and non-recurrence group in comparison with the other biological factors, whereas it was overexpressed in the clusters that discriminate high risk and low risk stage I groups in comparison with the others biological factors. An in vitro study found an inhibitory action of adiponectin on EC cells proliferation, suggesting a protective effect of adiponectin [25]. Adiponectin is a circulating factor and the study of its receptor in vivo would have been more accurate. A recent study found that underexpression of adipoR was associated with advanced stage, myometrial invasion and positive LNs [27]. Underexpression of RBP4 in the clusters could not be interpreted regarding the lack of data in the literature. Therefore, further research should focus on this obesity related marker, which had been identified as a link between insulinoresistance and adipose tissue by downregulating GLUT4 [21].

## 4. Material and Methods

### 4.1. Patients

**Figure 4:**
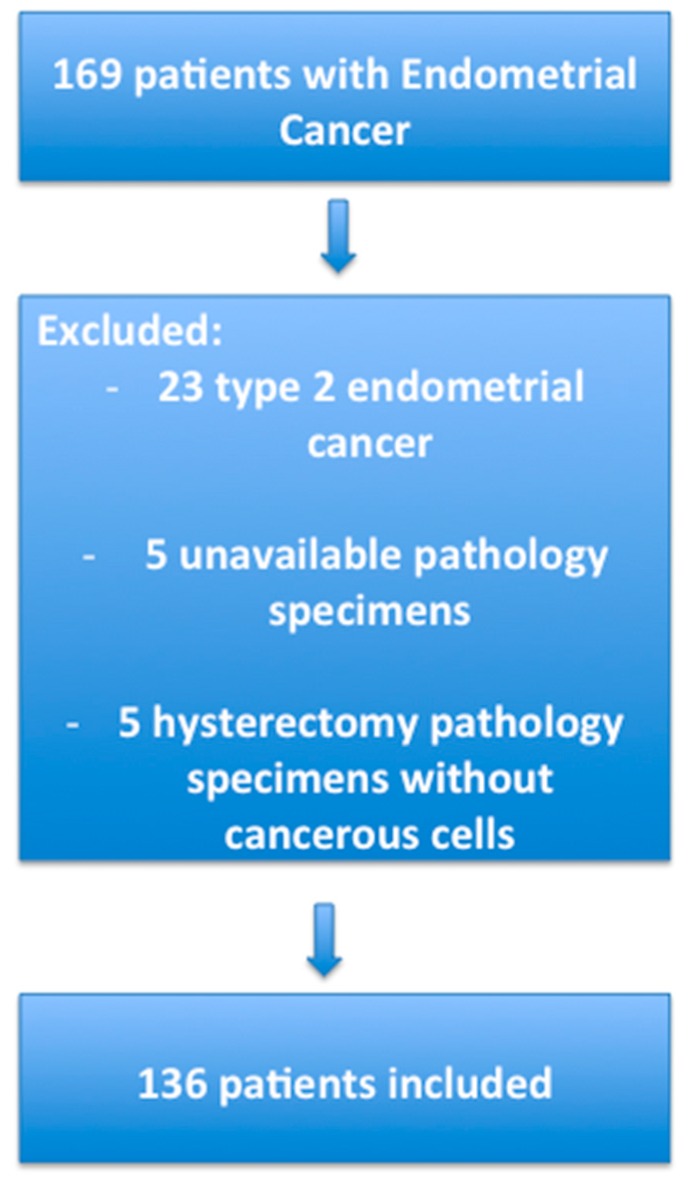
Flow chart of patients included in the study.

In this retrospective study, we selected all consecutive patients who had a total hysterectomy for type 1 EC between 1 January 2000 and 4 November 2013 in the Department of Gynecology and Obstetrics, Creteil University Hospital, France. Type 1 EC is defined by to the European Society of Medical Oncology (ESMO) group as endometrioid histological subtype. Exclusion criteria were type 2 EC, unavailable pathology specimens or absence of cancerous cells on hysterectomy pathology specimens. The flow chart of the patients included in the study is presented in Figure 4. Clinical data have been reported on an Excel database, including age, BMI, gestity, parity, menopausal status, hormonal treatments, history of diabetes, hypertension, dyslipidemia, familial history of cancer, radiological data (computed tomography, sonography, and magnetic resonance imaging), pathological data, FIGO stage, adjuvant treatment, progression free survival, and overall survival data. We used the classification from the International Federation of Gynecology and Obstetrics (FIGO) 2009. Patients were clinically followed every 4 months during the first three years then every six months up to five years then every year according to our national guidelines. The mean follow up was 41 months. Mean progression free survival and overall survival were 36.9 and 40.7 months, respectively. The study protocol was approved by the Ethics Committee of Paris X, France (14 January 2015; No. 2015-01-03).

### 4.2. Immunohistochemistry

We conducted IHC analysis from hysterectomy specimen of 136 patients with type 1 endometrial cancer. We used archive paraffin-embedded blocks of formalin-fixed hysterectomy pathology specimens processed by routine pathology. Tissues were fixed in formalin (10%) and then processed as paraffin blocks. Four micron-thick sections of formalin-fixed tissues were deparaffinized in a xylene substitute (EZ prep^®^) and rehydrated at 75 °C. The sections were immunostained using the Ventana Benchmark GX^®^ automated immunohistochemistry system (Optiview™ and Ultraview™, Universal DAB-Ventana^®^).

For IHC, the pathologists (IA and DM) chose the slides after H&E staining according to the following criteria: (i) significant tumor area; (ii) without necrosis; (iii) without artefacts of formalin-fixation; and (iv) without calcospherites. Indeed, we used one slide per antibody per patient. All slides came from the same block for one patient and were consecutive to analyze the same area.

### 4.3. Adipokines and Hormonal Receptors Immunostaining

We used a rabbit monoclonal antibodies directed against ER (1/100, SP1, MMFrance^®^, Brignais, France), PR (1/100, SP2, MMFrance^®^, Brignais, France) and SPARC (1/50, D10F10, 8725S, Ozyme^®^, Montigny-Le-Bretonneux, France). We used a mouse monoclonal antibodies for adiponectin (1/100, 1B2, TA503801, Origene^®^, Rockville, MD, USA), RBP4 (1/100, 4D9, LS-B6142-50, LifeSpan Bioscience^®^, Seattle, WA, USA), IL-6 (1/100, ab9324, Abcam^®^, Cambridge, UK), TNF α (1/100, 6C10, LS-B7086-50, LifeSpan Bioscience^®^, Seattle, WA, USA). An antigen retrieval procedure was run including an incubation at 95 or 100 °C with CC1 Ventana^®^ for 32 min for adiponectin, RBP4, IL-6 and SPARC or 60 min for ER, PR and TNF α. This automated procedure is based on an indirect biotin-avidin system. A universal biotinylated immunoglobulin was used as a secondary antibody, 3,3-diaminobenzidine as the substrate and hematoxylin as the counterstain. Positive controls were sections of human breast tissue for ER and PR, thyroid carcinoma for adiponectin (according to the manufacturer’s instructions), Langerhans islets for RBP4 (according to the manufacturer’s instructions), muscularis mucosae of vessels for IL-6 (according to the manufacturer’s instructions), endothelium for SPARC (according to the manufacturer’s instructions), lymphocytes, and neutrophils for TNF α (according to the manufacturer’s instructions). Signal was amplified for TNF α (Amplification kit Ventana^®^). Positive controls were breast adenocarcinoma for IL-6, thyroid carcinoma for adiponectine, pancreas for RBP4, liver for TNF α, endothelium for SPARC, and breast cancer for ER and PR.

### 4.4. Analysis of the Immunohistochemical Results

Immunohistochemical analysis was performed by one researcher (JU) under the supervision of two pathologists (DM and IAA) but no interrater comparison was made. Immunostainings were interpreted on the whole leaf at ×5, ×10 and ×20 magnification. We analyzed both the central part and the invading front which were present in all slides. For ER, PR, TNF α and RBP4, the intensity of staining was analyzed using the HSCORE: ΣPi × (*i* + 1), where *i* is the intensity of staining varying from 1 to 3 and Pi is the percentage of stained cells [29]. For example if 30% of cells have positive staining with intensity 1 and 70% of cells have positive staining with intensity 3, the HSCORE is 30 × 1 + 70 × 3 = 240 A composite score was developed by two pathologists (DM and IAA) to analyze the adiponectin staining: the percentage of stained cells was multiplied by the type of stained stromal cells to which we attributed a code (1 for vessels, 2 for fibroblasts). A composite score was also developed by the two pathologists (DM and IAA) to analyze IL-6 and SPARC staining; IL-6 and SPARC were stained in the tumoral cells and in the stromal cells so we attributed a “tumoral HSCORE” based on a regular HSCORE [29] and a “stromal HSCORE,” which was a modified HSCORE to which we multiplied by 1 if the vessels were majority stained or by 2 if the fibroblasts were majority stained.

### 4.5. Selection of the Groups

Expression of adipokines and hormonal receptors was evaluated in the general population (136 patients). We then compared the obese group (55 patients, 40.4%) and non-obese group (81 patients, 59.6%), and finally, we compared their expression in three prognosis groups in obese population.

The first prognostic group included the patients with recurrence (vaginal, pelvic, lymph nodes, or general) in comparison with a no recurrence group. The second prognostic group included the patients with positive lymph nodes (LNs) (from pelvic and/or para aortic lymphadenectomy) in comparison with a negative LNs group. The third prognostic group included the patients with high risk stage I tumor defined by FIGO stage IB and histological grade 3 or lymphovascular space involvement (LVSI) in comparison with a low risk stage I group defined by stage IA and histological grade 1, 2, or 3, or stage IB and histological grade 1 or 2 without LVSI. The low-risk stage I group encompassed the low and intermediate risk stage I defined by the European Society for Medical Oncology (ESMO) 2013 guidelines [35], which we added patients without LVSI, in order to simplify the statistical analysis.

### 4.6. Statistical Analysis

Quantitative expression of protein is expressed in mean value with standard deviation. Mean values in both groups were compared using *t*-test, for normal distribution, or non-parametric Wilcoxon test, as appropriate. We used two sided tests and a *p*-value <0.05 was considered as significant.

### 4.7. Supervised Clustering

The algorithm previously described by Dettling et al. was used for the supervised clustering [36]. The aim of this supervised algorithm is to identify protein clusters that are strongly associated with a supervised categorical response y (obese and non-obese) i.e., whose average expression profile has great potential for explaining the response (patients groups discrimination), given a small number of sample tissues with the expression activities of multiple proteins. The difficulty was that we knew neither the cluster size, nor the number of clusters (*q*). This method uses a clustering criterion, *S*, corresponding to a (penalized) goodness-of-fit measure from a penalized logistic regression analysis. First, the expression value of every protein is standardized to zero mean and unit variance. Variable selection and grouping are done by a stepwise forward search i.e., by trying each protein and increasing the group by the protein that optimizes the criterion *S*. After each forward search, it continues by means of a backward pruning step to root out proteins that have been incorrectly added to the group at the earlier forward stages. When the cluster can no longer be improved, a new cluster is started. The grouping procedure is supervised since all the decisions are based on optimizing the criterion *S* that measures the ability of the groups to explain the variable response y. By computing the grouping criterion directly from multiple groups instead of single groups only, we could obtain the best interacting protein groups that explain the response y as an ensemble. This method is advantageous in that it allows overlapping groups and that the groups together contribute most in predicting the response *y*. Cluster centroids (i.e., mean expression of the co-expressed proteins within a cluster) can be interpreted as a protein signature that is strongly differentially expressed and carries substantial information about predicting *y*. We used a bootstrap method with 1000 replication to specify the confidence interval of misclassification rate.

This statistical method has been used to distinguish atypical hyperplasia and grade 1 endometrioid endometrial cancer [37] or atypical and non-atypical endometrial hyperplasia [38] based on immunohistochemical markers of endometrial tissue samples.

Data were managed with an Excel database (Microsoft, Redmond, WA, USA) and analyzed using R 2.15 software with the Supclust library, available online (R: A Language and Environment for Statistical Computing, R Development Core Team, 2.15, 2014, Available online: https://www.r-project.org/).

## 5. Conclusions

In this study, we found that intra-tumoral adipokines and hormonal receptors expression was not different in the obese population in comparison with the non-obese population. However, we identified clusters of intra-tumoral adipokines and hormonal receptors predictive of high risk stage I and recurrence groups in obese population with type 1 EC. Thus, obese patients with type 1 EC could be classified in different prognostic groups based on IHC data independently of their clinical features. Those results need to be confirmed in general population and on biopsy specimen in a way to balance treatment morbidities associated with obesity and oncologic prognosis.

## Figures and Tables

**Table 1:** Characteristics of general and obese population.

Characteristics	General Population	Obese Population	*p*
Total	136	55	-
Age: mean (SD)	67.8 (11.1)	66.1 (16.2)	0.34
Menopausal status	128 (94.1%)	52 (94.5%)	1
Hysterectomy with bilateral SO *	129 (94.8%)	52 (94.5%)	1
Hysterectomy with unilateral SO	5 (3.6%)	2 (3.6%)	
Hysterectomy without SO	2 (1.6%)	1 (1.9%)	
Lymphadenectomy			0.43
Pelvic	110 (80.8%)	41 (74.5%)	
Pelvic and para aortic	8 (5.8%)	3 (5.4%)	
Histological grade			0.86
1	76 (55.8%)	29 (52.7%)	
2	38 (25.7%)	17 (30.9%)	
3	22 (18.5%)	9 (16.4%)	
Lymphovascular space involvement (LVSI)			0.49
Yes	33 (24.2%)	17 (30.9%)	
No	80 (58.8%)	30 (54.5%)	
Data not available	23 (17%)	8 (14.6%)	
FIGO ** stage			0.95
I	107 (78.7%)	43 (78.1%)	
IA	61 (44.8%)	20 (36.3%)	
IB	46 (33.8%)	23 (41.8%)	
II	8 (5.8%)	3 (5.4%)	
III	17 (12.7%)	8 (14.5%)	
IV	4 (2.8%)	1 (2.0%)	
Recurrence	25 (18.3%)	13 (23.6%)	0.61
Positive lymph node	11 (8.0%)	5 (9.1%)	0.90
High risk stage I ***	11 (8.0%)	7 (12.7%)	0.89

* SO: salpingo oophorectomy. ** FIGO: International Federation of Gynecology and Obstetrics. *** High risk stage I: FIGO 2009 stage IB and histological grade 3 or lymphovascular space involvement.

**Table 2:** Protein clustering in recurrence vs. non recurrence groups in obese population.

Cluster	Proteins Overexpressed	Proteins Underexpressed
Cluster 1	Stromal IL-6	PR
		RBP4
		ER
Cluster 2	Stromal IL-6	PR
		RBP4
Cluster 3	Stromal IL-6	PR
		RBP4
	TNF α	ER
		Stromal adiponectin

**Table 3:** Protein clustering in high risk and low risk stage I groups in obese population.

Cluster	Proteins Overexpressed	Proteins Underexpressed
Cluster 1	Stromal adiponectin	PR
		ER
		Stromal SPARC
		RBP4
Cluster 2	Stromal adiponectin	Stromal SPARC
		PR
		RBP4
Cluster 3	Stromal adiponectin	Stromal SPARC
		RBP4
		PR
Cluster 4	Stromal adiponectin Stromal IL-6	Stromal SPARC
		RBP4
		PR
		ER
		SPARC

## Supplementary Materials

Supplementary materials can be found at www.mdpi.com/1422-0067/18/5/1055/s1.
